# Supplementary material for: The long-term relationship between oil price changes and economic growth from the perspective of the resource curse: An empirical study from Yemen
Source: PLoS One. 2024 Nov 21;19(11):e0313206. doi: 10.1371/journal.pone.0313206 (PMC11581271; doi:10.1371/journal.pone.0313206)
Supplement: Attachment — Submitted filename: Response letter for reviewer #1.docx [file pone.0313206.s002.docx]

***The Long-Term relationship between oil price changes and economic growth from the perspective of the resource curse: An empirical study from Yemen***

***Author's Response***

***The Long-Term relationship between oil price changes and economic growth from the perspective of the resource curse: An empirical study from Yemen***

Respected Editor

Thank you very much for the opportunity to revise and resubmit our research paper. We are particularly very grateful to all reviewers for their constructive and valuable comments. The paper has been carefully revised again to accommodate all your comments and suggestions. We hope this revised paper will now meet the standard of the PLOS ONE journal for publication.

Thank you again for the comments and suggestions.

Yours Truly,

The Authors


**REVIEWER #1:
COMMENTS (# 1):**

The paper uses language that indicates that they are presenting a causal relationship, such as “influence”, “impact”, and “affect” in the abstract. This type of language continues throughout the paper. However, there does not seem to be anything causal about the relationship that is being studied here. The authors have no way to prove that the changes in oil prices are directly affecting GDP, as many other outside elements including changes in the Yemeni policy space may be happening at the same time. One could argue, in some way, that the oil prices are exogenous to Yemen, though they are contributing in some way through their own production and export of oil. It is also possible that Yemen is, for example, enacting policies that affect both oil prices and GDP, with the effect on GDP being slightly delayed. If the authors want to present a causal relationship, they need to make some argument that what they are studying is causal and probably use some form of statistical identification to do so.

*Response:*
Thank you for your valuable comments and feedback on our manuscript. We appreciate the time and effort you have invested in reviewing our work.

We acknowledge that our use of certain terminology may have unintentionally suggested that our study was focused on establishing a causal effect relationship between the variables under investigation. Our primary objective was to examine the long-term relationships between oil price changes and economic growth using the ARDL model, which is designed for this purpose. The robustness checks conducted using GMM, GLM, and RLS also support the presence of long-term relationships between the variables. We did not intend to imply a causal effect relationship, and we apologize for any confusion this may have caused.

Based on your insightful comments, we have made linguistic adjustments to our title and abstract to more accurately reflect the purpose of our study, which is to explore the long-term relationships rather than causal effects.

[Please refer to the title, abstract, and lines (54, 295, 346, 427, 430, 440, 442, 446, and 472)]

In response to your valuable suggestion, we have conducted an additional analysis to explore potential causal relationships between the variables. The results of this analysis have been included in the revised manuscript to provide a clearer picture and ensure that our use of terminology is consistent with our findings.

Thank you again for your constructive feedback. We believe these revisions enhance the clarity and rigor of our study. We hope the new version will meet your kind expectations.

[Please refer to the lines (500 to 509)]

**COMMENT (# 2):**

It is unclear what the paper’s contribution is relative to the literature. As the authors say on page 7, “…Victor & Ogbonna (2018) have proven in their study that oil price fluctuations have a positive impact on economic growth…”. If that has already been shown, what is the additional value of this paper?

I will list three areas that seemed like they could have been contributions, but did not quite make it in terms of execution:

a. The paper talks about studying price fluctuations, but what actually seems to be studied is price changes. When I hear price fluctuations, I think of some measure of variance that is independent of whether the price is going up or down. However, the independent variable here is price changes, which have a directional element. It seems unsurprising that, in an oil producing country, when the price of oil goes up the GDP goes up (and seems to also have been studied quite extensively previously). What I think would be more interesting is to study how variability in oil prices affects GDP in an oil-exporting country.

b. The paper points to Yemen being a “burgeoning oil-exporting nation” as an area of contribution. However, the authors never outline how they mean burgeoning. Do they mean that their discovery and export of oil is relatively recent (as seems to be suggested in the paper)? Or do they mean that the country is burgeoning in some other way, like increasing GDP or economic development? Furthermore, the authors do not outline why this burgeoning is interesting in terms of the relationship between oil prices and GDP relative to what is already studied in the other literature. I would think that to study this aspect well, the authors would also need pre-oil data for Yemen.

c. The authors also talk about oil rents as an outcome. However, at no point do they define what oil rents are, why they would relate to GDP, or why this is interesting to study.

*Response:*
Thank you for your insightful comments and for taking the time to review our manuscript. We appreciate your feedback and would like to address the concerns raised regarding the contribution of our paper relative to the existing literature.

While Victor & Ogbonna (2018) have indeed shown a positive impact of oil price fluctuations on economic growth, our study focuses on the unique context of Yemen, a burgeoning oil-exporting nation with distinct economic dynamics. Yemen's socio-economic environment, policy framework, and developmental challenges differ significantly from those of the countries examined in previous studies.

Our study employs the auto-regressive distributed lag (ARDL) model, supplemented with robustness checks using GLM, RLS, and GMM. These methodologies are particularly suited for examining the long-term relationships in Yemen's context. The robustness checks validate the consistency of our findings, adding to the methodological rigor of our study.

Our research offers new insights into the negative long-term relationship between oil rents and economic growth in Yemen. Our recommendations are tailored to Yemen's specific context and are aimed at reducing the country's dependence on oil, thus promoting sustainable economic growth.

Based on your valuable comment, we have included a detailed explanation and comparison with existing literature to highlight the unique context of Yemen's economic situation and the new insights and contributions our study provides.

[Please refer to the introduction section in lines (126 -152)]

A. Regarding the important issues that you referred to in comment (a), while we appreciate these important comments, we would like to clarify as follows:

We acknowledge the distinction you made between price fluctuations and price changes. In our study, we aimed to investigate the impact of oil price volatility on economic growth. However, we used the term "price fluctuations" interchangeably with "price changes," which may have caused some confusion. Our primary focus was on the directional changes in oil prices (i.e., increases and decreases) and their subsequent impact on GDP, rather than on the variability or variance of these prices.

The rationale behind examining price changes, rather than variability, is rooted in the economic realities faced by oil-exporting countries like Yemen. Directional price changes directly influence national income, government revenue, and investment capacity due to the reliance on oil exports. This approach allows us to capture the immediate and tangible effects of oil price movements on economic growth. While variability in prices could be an interesting angle, our study specifically aimed to understand how price increases and decreases affect GDP, given the significant role of oil revenues in Yemen’s economy.

While it might seem intuitive that rising oil prices lead to economic growth in an oil-exporting country, our study contributes by quantifying this relationship using the ARDL model over an extended period (1990-2019). Furthermore, we provide empirical evidence on how oil rents, another critical variable, negatively impact economic growth, offering a nuanced understanding of the resource curse phenomenon in Yemen. These findings are crucial for policymakers in devising strategies to mitigate the adverse effects of oil dependency.

We agree that examining the impact of price variability (variance) on GDP could provide additional insights. This could form the basis for future research, expanding the scope of our current study. However, the focus on price changes in our paper is deliberate, aimed at addressing immediate policy-relevant questions regarding how oil price movements influence economic growth dynamics in Yemen.

In light of your comments, we have revised the manuscript to ensure clearer terminology and have explicitly stated our focus on price changes. Additionally, we acknowledge the potential for future studies to explore the impact of price variability on economic growth.

[Please refer to the lines (54, 295, 346, 427, 430, 440, 442, 446, and 472) and future studies section (591-593)]

Please feel free for any suggestion from you that would help improve our work. Thank you for your time and consideration. We are really grateful for your sincere efforts to improve the overall quality of our work.

B. Regarding the important issues that you referred to in comment (b), while we appreciate these important comments, we would like to clarify as follows:

In our paper, we describe Yemen as a “burgeoning oil-exporting nation” to indicate its relatively recent entry and growth in the global oil market compared to long-established oil-exporting countries. Yemen began exporting oil in the late 1980s, with significant development in oil production and export infrastructure occurring in the 1990s. This period marks Yemen’s transition to becoming a notable player in the oil market, which contrasts with countries that have been major oil exporters for many decades. Therefore, by "burgeoning," we mean that Yemen’s oil sector is comparatively young and rapidly developing.

The burgeoning status of Yemen as an oil-exporting nation is particularly interesting because it allows us to examine the impact of oil price Changes on an economy at a different stage of development compared to more mature oil-exporting countries. Most existing literature focuses on well-established oil economies with extensive historical data, whereas Yemen provides a unique case study of how a newer entrant into the oil market navigates economic growth amid oil price volatility. This distinction is crucial as it adds diversity to the understanding of the resource curse and its effects on different types of economies.

While we agree that pre-oil data could provide additional insights, Yemen's formal oil exportation and significant economic structuring around oil began in the late 1980s. Consequently, relevant pre-oil economic data is scarce or not systematically recorded, limiting its utility for robust econometric analysis. Our study focuses on the available post-oil data from 1990 to 2019, which captures the period when Yemen’s economy has been significantly influenced by oil exports. This timeframe allows us to study the relationship between oil price Changes and economic growth during the most impactful period of Yemen’s oil-driven economic development.

Our study fills a gap in the existing literature by exploring the dynamics of oil price Changes and economic growth in a relatively new oil-exporting country. Unlike established oil economies with extensive historical data, Yemen provides a contemporary context to understand how newer oil exporters might experience and adapt to oil price volatility. This perspective can offer valuable insights for policymakers in similar burgeoning oil-exporting countries, helping them to design strategies that mitigate negative impacts and harness potential benefits of their natural resources.

In light of your value comments, we have revised the manuscript to incorporate these explanations and ensure that our arguments are clear and well-supported.

[Please refer to the introduction section (118-125)]

Please feel free for any suggestion from you that would help improve our work. Thank you for your time and consideration. We are really grateful for your sincere efforts to improve the overall quality of our work.

C. Regarding the important issues that you referred to in comment (c), while we appreciate these important comments, we would like to clarify as follows:

Oil rents are defined as the difference between the value of crude oil production at world prices and the total costs of production. This measure captures the revenue generated from oil extraction after accounting for production costs, representing a crucial component of national income for oil-exporting countries like Yemen.

Oil rents are directly related to GDP as they constitute a significant portion of the national income for oil-exporting countries. The revenues generated from oil rents can have substantial implications for economic growth, government spending, and overall economic stability. High oil rents can lead to increased government revenue, which can be invested in infrastructure, public services, and other development projects, thereby influencing GDP.

Studying oil rents is particularly interesting in the context of the resource curse theory, which suggests that countries with abundant natural resources often experience less economic growth compared to those with fewer resources. Understanding the relationship between oil rents and GDP can provide insights into how reliance on natural resource revenues impacts economic development. In the case of Yemen, examining oil rents helps to assess whether the country is experiencing the resource curse and how fluctuations in oil rents affect its economic growth.

We hope these clarifications address your concerns, based on your valuable comments, we have revised the manuscript and added the necessary explanations to address these issues that raised your concerns and ensured that our arguments became clear and well supported.

[Please refer to the data section (275-283)]

**COMMENT (# 3):**

I am also concerned with the empirical specification used in this paper for multiple reasons:

A. First, I am concerned about the controls that are being used. The authors argue that controls such as the exchange rate and the inflation rate are used because they affect GDP. However, they also argue in the introduction and literature section that changes in oil prices affect these things as well. My understanding is that if you are trying to capture the relationship between oil prices and GDP here, you should not include as controls things that could be mechanisms of that relationship.

B. In equation 6, the sums go from i=0 to i=q. Shouldn’t that actually be from i=2 to i=q, since for i=1 they are captured by the beta terms and for i=0 they are the concurrent changes? I’m assuming that this is an error in the manuscript, rather than what was actually done in the analysis since the authors would have gotten very wacky things in Table 8 otherwise.

*Response:*
Dear reviewer, thank you for your valuable feedback on our paper, we appreciate the time and effort you have dedicated to reviewing our work.

A. Regarding the important issues that you referred to in comment (a), while we appreciate these important comments, we would like to clarify as follows:

We included the exchange rate and inflation rate as control variables in our model because they are key macroeconomic indicators that can influence GDP. While it is true that changes in oil prices can affect these variables, their inclusion helps to isolate the specific impact of oil price changes on GDP by accounting for these broader economic factors.

Our primary objective is to understand the relationship between oil price changes and GDP. By controlling for the exchange rate and inflation rate, we aim to capture the direct effect of oil price changes on economic growth, while mitigating the influence of these intermediary variables. This approach is supported by econometric theory, which suggests that including relevant controls can help to obtain more accurate estimates of the variable of interest (in this case, oil prices) on the dependent variable (GDP).

Numerous empirical studies have demonstrated the importance of including control variables that can potentially mediate the relationship between the main explanatory variable and the dependent variable. For instance, studies on the impact of oil prices on economic performance often include macroeconomic indicators such as exchange rates and inflation rates to account for their indirect effects and to enhance the robustness of the results.

We hope these clarifications address your concerns and underscore the robustness and validity of our research methodology. We have revised our manuscript to incorporate these explanations to ensure that our arguments are clear and well-supported.

[Please refer to the data section in lines (284-291)]

Thank you again for your constructive feedback. We are committed to enhancing our paper and believe these revisions will significantly improve its clarity and impact.

B. Regarding the important issues that you referred to in comment (b), while we appreciate these important comments, we would like to clarify as follows:

Thank you for your meticulous review and insightful comments on our manuscript.

You are correct in your observation regarding Equation 6. The sums should indeed go from (i=2) to (i=q), as for (i=1) they are captured by the beta terms and for (i=0) they represent the concurrent changes. This was a clerical error in the manuscript and not a reflection of what was actually done in the analysis.

The results presented in Table 8 confirm that our model specification is correct. If there had been an error in the summation indices, it would have led to anomalous results, as you rightly pointed out. However, the consistency and robustness of our findings across various tests (GLM, RLS, GMM) indicate that the model was specified and implemented correctly in the analysis.

We have modified the equation 6 in the manuscript according to your comment to accurately reflect the intended analysis. We appreciate your attention to detail and are grateful for your feedback, which has helped improve the clarity and accuracy of our work.

[Please refer to the line (355) equations 6]

**COMMENT (# 4):**

Finally, I believe the draft would be strongly helped by some rewriting and editing. For example, there appears to be a random paragraph break between the first two paragraphs of the introduction and the first sentence of Section 3.1 seems to have a wrong word (“country”?) that makes it difficult to understand. More importantly, the introduction and literature sections can be reworked to better outline the research question and emphasize the contribution of this paper relative to the literature. Currently, a lot of words are spent outlining other papers’ theories and results without indicating how they relate to the current paper. A stronger introduction and literature review would outline the motivation for the question at hand, very clearly state the exact research question being studied in this paper, and then summarize the prior literature in relation to how it sets up but does not answer the question of the current paper. This will give the readers and possibly also the authors greater clarity on what this paper is and is not able to do.

*Response:*
Thank you for your thorough and constructive feedback on our manuscript. We have carefully addressed all the issues you mentioned to improve the clarity and quality of our paper.

We have thoroughly reviewed and edited the manuscript to ensure coherence and eliminate any random paragraph breaks. Specifically, we corrected the paragraph break between the first two paragraphs of the introduction and clarified the wording in the first sentence of Section 3.1 to ensure it accurately conveys our intended meaning.

We have reworked the introduction to better outline the research question and emphasize our paper's contribution relative to the existing literature. The revised introduction now clearly states the motivation for our research, explicitly outlines the exact research question we are addressing, and succinctly summarizes the prior literature in a way that highlights how it sets up but does not answer the question posed by our study.

We believe these revisions significantly enhance the readability and impact of our paper. We hope this revised version will be well received. We are grateful for your comments, which have greatly contributed to improving the quality of our paper. We remain open to any further amendments or clarifications that may be required.

Thank you again for your valuable feedback.

[Please refer to the lines (56-117) and (160-211)]
