# Supplementary material for: The long-term relationship between oil price changes and economic growth from the perspective of the resource curse: An empirical study from Yemen
Source: PLoS One. 2024 Nov 21;19(11):e0313206. doi: 10.1371/journal.pone.0313206 (PMC11581271; doi:10.1371/journal.pone.0313206)
Supplement: Attachment — Submitted filename: response letter for reviewer No(2).docx [file pone.0313206.s003.docx]

***The Long-Term relationship between oil price changes and economic growth from the perspective of the resource curse: An empirical study from Yemen***

***Author's Response***

***The Long-Term relationship between oil price changes and economic growth from the perspective of the resource curse: An empirical study from Yemen***

Respected Editor

Thank you very much for the opportunity to revise and resubmit our research paper. We are particularly very grateful to all reviewers for their constructive and valuable comments. The paper has been carefully revised again to accommodate all your comments and suggestions. We hope this revised paper will now meet the standard of the PLOS ONE journal for publication.

Thank you again for the comments and suggestions.

Yours Truly,

The Authors


**REVIEWER #2:
COMMENTS (# 1):**

Table 10 should be designed in consistent with other tables in the manuscript. The reference section should be written inline with Plos one guideline.

*Response:*
Thank you for your valuable comments and feedback on our manuscript. We appreciate the time and effort you have invested in reviewing our work. Thank you for your thoughtful feedback and for acknowledging the clarifications we made regarding our second revision. We appreciate the opportunity to further refine our work and address your remaining concerns.

Table 10 have been designed in consistent with other tables in the manuscript. [Please refer to table 10]

The reference section also has been changed to be consistent with Plos one guideline. [Please refer to references section]

Thank you again and hope this revised revision will now meet your expectation.
